# Does prolonged prone position affect intracranial pressure? prospective observational study employing Optic nerve sheath diameter measurements

**DOI:** 10.1186/s12871-023-02037-9

**Published:** 2023-03-14

**Authors:** Ufuk Demir, Öztürk Taşkın, Ayşe Yılmaz, Veysel G. Soylu, Zahide Doğanay

**Affiliations:** 1 Department of Anesthesiology and Reanimation, Faculty of Medicine, Kastamonu University, 37100 Kastamonu, Turkey; 2 Department of Intensive Care, Faculty of Medicine, Kastamonu University, Kastamonu, Turkey

**Keywords:** Prone position, Optic nerve sheath diameter, ARDS, Intracranial pressure, Neuroimaging, Intracranial pressure monitoring

## Abstract

**Background:**

Our aim in this observational prospective study is to determine whether the prone position has an effect on intracranial pressure, by performing ultrasound-guided ONSD (Optic Nerve Sheath Diameter) measurements in patients with acute respiratory distress syndrome (ARDS) ventilated in the prone position.

**Methods:**

Patients hospitalized in the intensive care unit with a diagnosis of ARDS who were placed in the prone position for 24 h during their treatment were included in the study. Standardized sedation and neuromuscular blockade were applied to all patients in the prone position. Mechanical ventilation settings were standardized. Demographic data and patients’ pCO_2_, pO_2_, PaO_2_/FiO_2_, SpO_2_, right and left ONSD data, and complications were recorded at certain times over 24 h.

**Results:**

The evaluation of 24-hour prone-position data of patients with ARDS showed no significant increase in ONSD. There was no significant difference in pCO_2_ values either. PaO_2_/FiO_2_ and pO_2_ values demonstrated significant cumulative increases at all times. Post-prone SPO2 values at the 8th hour and later were significantly higher when compared to baseline (p < 0.001).

**Conclusion:**

As a result of this study, it appears that the prone position does not increase intracranial pressure during the first 24 h and can be safely utilized, given the administration of appropriate sedation, neuromuscular blockade, and mechanical ventilation strategy. ONSD measurements may increase the safety of monitoring in patients ventilated in the prone position.

## Introduction

Coronavirus disease 2019 (COVID-19) is an important new cause of ARDS. In the current COVID-19 pandemic, the prone position for ARDS has been widely adopted by clinicians and is even used before intubation in spontaneously breathing patients [1, 2]. In hypoxemic patients with ARDS, there are studies which have shown that it provides various benefits and reduces mortality, especially when applied for a longer period of time [3, 4].

Many complications have been reported in patients during the prone position and when transitioning to the prone position. Some researchers have reported increased intracranial pressure (ICP) as a complication of the prone position [5–7]. However, there is insufficient data regarding the development of ICP and its clinical significance.

Although invasive methods (intraventricular and intraparenchymal monitors) are still the gold standard in the diagnosis and follow-up of increased ICP, these approaches may cause major complications, such as hemorrhage and infection [8]. Non-invasive alternative methods recommended for the assessment of ICP include the following: measurement of changes in cranial computed tomography (CT) and brain magnetic resonance imaging (MRI), transcranial doppler, tympanic membrane displacement, intraocular pressure, venous ophthalmodynamometry and ONSD [8].

The measurement of ONSD is established to be effective in detecting ICP increase due to the reflection of the pressure in the subarachnoid space and resultant changes in the optic nerve sheath. Studies comparing ONSD measurements with invasively measured ICP have reported that ONSD values are predictive for the detection of increased ICP, with analyses showing 95% sensitivity and 92% specificity. In addition, ONSD can be measured using CT and MRI, but the accuracies of measurements made with these two methods are lower [9, 10].

Our aim in this observational prospective study was to evaluate the effect of prone position on ICP by performing ultrasound-guided ONSD measurements during prone positioning in patients with ARDS diagnosis, and if there is an effect, to determine when the prone position begins to influence ICP.

## Materials and methods

This study was planned in accordance with the Helsinki Declaration, the national Patient Rights Regulation, and all relevant ethical principles. Approval for the study was obtained from the local ethics committee.

Between March–August 2022, patients admitted to the intensive care unit with a diagnosis of ARDS who were scheduled to be placed in the prone position for treatment were assessed for inclusion. Subjects aged 18–75 years with normocarbia (pCO_2_: 30–45 mmHg), patients without eye-related disease, neurological disease or previous incident of increased ICP, and those without a history of neuro-ophthalmic surgery were enrolled into the study.

Patients who did not meet these criteria or refused participation (patient or legal representative) were excluded from the study. Relatives of all patients included in the study were informed about the study and written informed consent for study inclusion was obtained from relatives or legal representatives.

Throughout our study, patients with a diagnosis of ARDS who had a PaO_2_/FiO_2_ value of < 150 mmHg were placed in the prone position for 24 h. Sedation and neuromuscular blockade were applied to all patients during the prone position. Mechanical ventilation settings were standardized.

Demographic data of the patients were recorded. In addition, patients’ pCO_2_, pO_2_, PaO_2_/FiO_2_, SpO_2_ and right ONSD and left ONSD data were recorded at certain time-points detailed below. Complications that developed during the follow-up period were also recorded.

Data recording times were: T_0_, T_1_, T_4_, T_8_, T_12_, T_16_, T_20_ and T_24_ (numbered with respect to hours from transition to the prone position). T_0_: baseline, obtained immediately before transition to the prone position. T_1_, T_4_, T_8_, T_12_, T_16_, T_20_ and T_24_: time after prone positioning (T_hour_).

### Sedation, neuromuscular blockade and mechanical ventilation

Midazolam (bolus dose: 2 mg, maintenance dose: 0.05 mg/kg/h), propofol (bolus dose: 2 mg/kg, maintenance dose: 2–3 mg/kg/h), and rocuronium (bolus dose: 0.6 mg/kg, maintenance dose: 0.3 mg/kg/h) infusions were administered to achieve sedation and neuromuscular blockade. Bolus doses were administered 1 h before transition into the prone position, and then infusions were initiated. The patients were placed in the prone position 1 h after the bolus dose. Mechanical ventilation settings were set as follows: 6 ml/kg for estimated body weight, Pplateau < 30 mmHg, and 10 mmHg PEEP.

### ONSD measurement

**Fig. 1:**
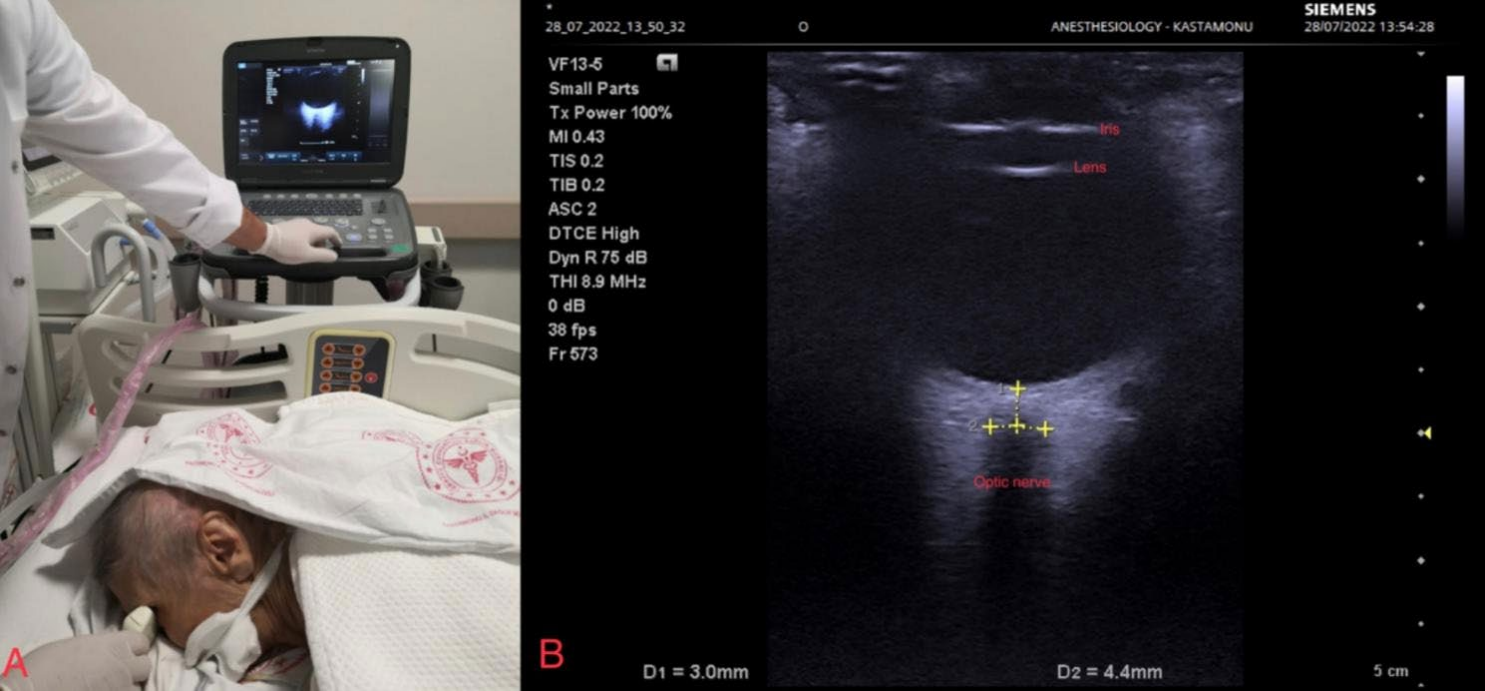
**A**- ONSD measurement in the prone position, **B**- ONSD measurement technique

All measurements were performed together by two experienced anesthesiologists while the patient was in the supine position at T0, and in the prone position thereafter. Patients’ head orientations remained in the neutral position throughout prone positioning. Only during ONSD measurement, the head position was slightly turned to the right or left, with respect to the eye undergoing ONSD measurement. Immediately after ONSD measurement, the heads of the patients were returned to the neutral position. The 13.5-MHz linear probe of the ultrasound device (Siemens, Mountain View, CA, USA) was used for the measurement. Gel was applied to the closed eyelids of the patients. The transducer was placed on the eyelids, taking care not to apply pressure. The depth on the USG device was set to 4–5 cm. With this adjustment, when focusing on the retrobulbar area, optimum contrast was achieved between the optic nerve sheath and the periorbital adipose tissue, and the sheath diameter perpendicular to this axis was measured by a 3-mm displacement from the posterior of the optic disc in the longitudinal axis. Two measurements were performed for each optic nerve. The first was carried out on the transverse plane with the probe placed horizontally. The second was performed on the sagittal plane during which the probe was placed vertically (Fig. 1). The final ONSD value was calculated by averaging these measurements and these values were recorded for each eye separately.

### Statistical analysis

According to descriptive statistics (effect size = 1.26, mean difference = 0.36, standard deviation of effects = 0.32) obtained from the study by Geng et al. [11], a sample size of 13 was determined to achieve 90% power for the two-sided 0.05 significance level. Sample size was calculated by using repeated measures analysis of variances power analysis via PASS software (Hintze, J. (2011). PASS 11. NCSS, LLC. Kaysville, Utah, USA. www.ncss.com.).

The IBM SPSS Statistics for Windows (Version: 22.0, IBM Corp., NY, USA) package program was used for statistical analysis. The Shapiro-Wilk test was used to determine whether continuous variables were normally distributed. Categorical variables are given as frequency (percentage), continuous variables are given as mean ± standard deviation or median (interquartile range, IQR) depending on normality of distribution. Normally distributed variables were analyzed with the repeated measures analysis of variance (ANOVA). Non-normally distributed variables were analyzed with the Friedman’s analysis of variance by ranks. Post-hoc analyses were adjusted with the Bonferroni correction. A p value of < 0.05 was considered significant in all statistics.

## Results

**Fig. 2:**
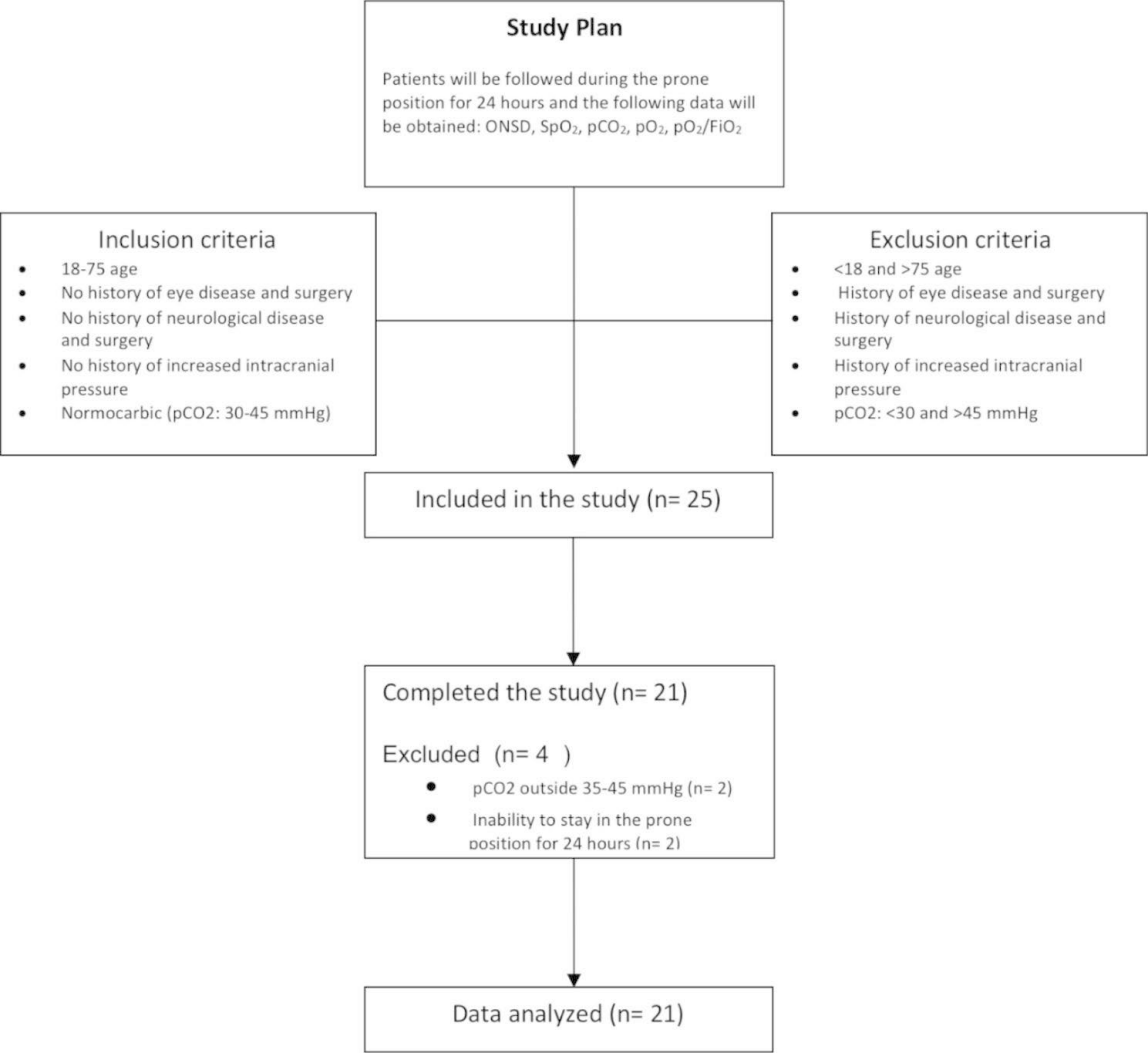
Flowchart of study

Twenty-five patients were assessed for inclusion. Four patients were excluded from the study, including two patients whose pCO_2_ value did not remain within the range of 35–45 mmHg during follow-up, and two patients who could not stay in the prone position for 24 h due to hemodynamic instability. The study was completed with 21 patients (Fig. 2).

Of the participants in our study, 9 (42.9%) were female, 12 (57.1%) were male, and the mean age was 55.48 ± 10.68 years. Comorbidities were as follows: diabetes mellitus in 5 (23.8%), hypertension in 8 (38.1%), and chronic obstructive pulmonary disease in 3 (14.3%) patients. With regard to prone position-related complications, three (14.3%) cases of facial edema were recorded (Table 1).

**Table 1:** Demographic data

Parameters	The number of patients (%)
Sex	
Female	9 (42.86%)
Male	12 (57.14%)
Age (years)	55.48 ± 10.68
Comorbid Disease	
No	5 (23.81%)
Diabetes Mellitus	5 (23.81%)
Hypertension	8 (38.10%)
Chronic Obstructive Pulmonary Disease	3 (14.29%)
Complication	
None	18 (85.71%)
Facial Edema	3 (14.29%)

Categorical variables are given as frequency (percentage). Age is given as mean ± standard deviation due to normality of distribution

The SpO_2_, pCO_2_, pO_2_ and PaO_2_/FiO_2_ data of the patients were recorded at all time points. Post-prone SPO2 values at the 8th hour and later were significantly higher when compared to baseline values (p < 0.001), the median (IQR) values increased to 94% (93–95%) at T_12_, T_16_, T_20_ and T_24_ from a baseline value of 81% (79–83%). Data for pCO_2_ showed similar values at all time-points. When the pO_2_ and PaO_2_/FiO_2_ data of the patients were compared, a cumulative significant increase was found in the comparison of each consecutive time-point (Table 2).

**Table 2:** SpO_2_, pCO_2_, pO_2_ and PaO_2_/FiO_2_ values measured at each time-point

	Baseline (T_0_)	Post-Prone 1 h (T_1_)	Post-Prone 4 h (T_4_)	Post-Prone 8 h (T_8_)	Post-Prone 12 h (T_12_)	Post-Prone 16 h (T_16_)	Post-Prone 20 h (T_20_)	Post-Prone 24 h (T_24_)	p
**SpO**_**2**_ ^**%**^	81 (79–83) ^a^	88 (87–90) ^ab^	92 (91–93) ^bc^	93 (92–94) ^c^	94 (93–94) ^c^	94 (93–95) ^c^	94 (93–95) ^c^	94 (93–95) ^c^	**< 0.001**
**Post-hoc** **p**	**T**_**0**_ **-T**_**1**_ **1.000**	**T**_**1**_ **-T**_**4**_ **0.709**	**T**_**4**_ **-T**_**8**_ **1.000**	**T**_**8**_ **-T**_**12**_ **1.000**	**T**_**12**_ **-T**_**16**_ **1.000**	**T**_**16**_ **-T**_**20**_ **1.000**	**T**_**20**_ **-T**_**24**_ **1.000**	**T**_**0**_ **-T**_**24**_ **< 0.001**	
**pCO**_**2**_ ^**mmHg**^	40 (38–41)	40 (37–41)	40 (38–42)	39 (37–41)	39 (39–41)	41 (39–42)	40 (39–41)	41 (40–42)	**0.378**
**Post-hoc** **p**	**T**_**0**_ **-T**_**1**_ **-**	**T**_**1**_ **-T**_**4**_ **-**	**T**_**4**_ **-T**_**8**_ **-**	**T**_**8**_ **-T**_**12**_ **-**	**T**_**12**_ **-T**_**16**_ **-**	**T**_**16**_ **-T**_**20**_ **-**	**T**_**20**_ **-T**_**24**_ **-**	**T**_**0**_ **-T**_**24**_ **-**	
**pO**_**2**_ ^**mmHg**^	68.05 ± 4.98 ^a^	72.90 ± 4.18 ^b^	81.38 ± 5.30 ^c^	90.67 ± 6.53 ^d^	103.62 ± 10.00 ^e^	116.62 ± 12.25 ^f^	126.71 ± 13.21 ^g^	139.43 ± 14.38 ^h^	**< 0.001**
**Post-hoc** **p**	**T**_**0**_ **-T**_**1**_ **< 0.001**	**T**_**1**_ **-T**_**4**_ **< 0.001**	**T**_**4**_ **-T**_**8**_ **< 0.001**	**T**_**8**_ **-T**_**12**_ **< 0.001**	**T**_**12**_ **-T**_**16**_ **< 0.001**	**T**_**16**_ **-T**_**20**_ **< 0.001**	**T**_**20**_ **-T**_**24**_ **< 0.001**	**T**_**0**_ **-T**_**24**_ **< 0.001**	
**PaO**_**2**_ **/FiO**_**2**_ ^**mmHg**^	84.67 ± 6.16 ^a^	90.76 ± 5.26 ^b^	101.29 ± 6.72 ^c^	112.90 ± 8.18 ^d^	129.14 ± 12.47 ^e^	145.48 ± 15.26 ^f^	158.00 ± 16.51 ^g^	174.00 ± 18.00 ^h^	**< 0.001**
**Post-hoc** **p**	**T**_**0**_ **-T**_**1**_ **< 0.001**	**T**_**1**_ **-T**_**4**_ **< 0.001**	**T**_**4**_ **-T**_**8**_ **< 0.001**	**T**_**8**_ **-T**_**12**_ **< 0.001**	**T**_**12**_ **-T**_**16**_ **< 0.001**	**T**_**16**_ **-T**_**20**_ **< 0.001**	**T**_**20**_ **-T**_**24**_ **< 0.001**	**T**_**0**_ **-T**_**24**_ **< 0.001**	

SpO_2_ and pCO_2_ are given as median (interquartile range) due to non-normality of distribution. These variables were analyzed with the Friedman’s analysis of variance by ranks. pO_2_ and PaO_2_/FiO_2_ are given as mean ± standard deviation due to normality of distribution. These variables were analyzed with the repeated measures analysis of variance. Post-hoc tests were adjusted by the Bonferroni correction. ^a−h^: Same letters denote the lack of statistically significant differences between the respective time-points

**Fig. 3:**
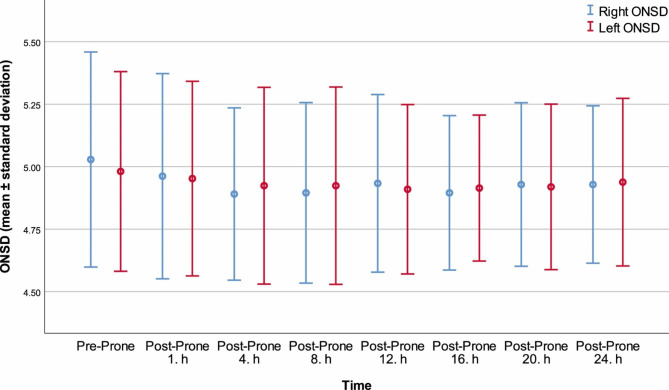
Right and left ONSD mean values during prone position

The ONSD data of the patients were recorded separately for the right and left eye at all time-points (Fig. 3). Comparison of consecutive ONSD values for each eye did not yield any significant differences (Table 3).

**Table 3:** Right and left ONSD values during prone position

	Baseline (T_0_)	Post-Prone 1 h (T_1_)	Post-Prone 4 h (T_4_)	Post-Prone 8 h (T_8_)	Post-Prone 12 h (T_12_)	Post-Prone 16 h (T_16_)	Post-Prone 20 h (T_20_)	Post-Prone 24 h (T_24_)	p
**Right ONSD** ^**mm**^	5.03 ± 0.43	4.96 ± 0.41	4.89 ± 0.34	4.90 ± 0.36	4.93 ± 0.36	4.90 ± 0.31	4.93 ± 0.33	4.93 ± 0.31	**0.182**
**Post-hoc** **p**	**T**_**0**_ **-T**_**1**_ **-**	**T**_**1**_ **-T**_**4**_ **-**	**T**_**4**_ **-T**_**8**_ **-**	**T**_**8**_ **-T**_**12**_ **-**	**T**_**12**_ **-T**_**16**_ **-**	**T**_**16**_ **-T**_**20**_ **-**	**T**_**20**_ **-T**_**24**_ **-**	**T**_**0**_ **-T**_**24**_ **-**	
**Left ONSD** ^**mm**^	4.98 ± 0.40	4.95 ± 0.39	4.92 ± 0.39	4.92 ± 0.39	4.91 ± 0.34	4.91 ± 0.29	4.92 ± 0.33	4.94 ± 0.34	**0.733**
**Post-hoc** **p**	**T**_**0**_ **-T**_**1**_ **-**	**T**_**1**_ **-T**_**4**_ **-**	**T**_**4**_ **-T**_**8**_ **-**	**T**_**8**_ **-T**_**12**_ **-**	**T**_**12**_ **-T**_**16**_ **-**	**T**_**16**_ **-T**_**20**_ **-**	**T**_**20**_ **-T**_**24**_ **-**	**T**_**0**_ **-T**_**24**_ **-**	

All ONSD values are given as mean ± standard deviation due to normality of distribution. These variables were analyzed with the repeated measures analysis of variance

## Discussion

This study analyzed 24 h of post-prone data concerning oxygenation and ICP (as determined via ONSD) in patients with ARDS. As a result of the analyses, no significant increase in ICP was observed throughout the first 24 h of prone positioning. The pCO_2_ values of patients also remained similar throughout this period; whereas, PaO_2_/FiO_2_ and pO_2_ values demonstrated a cumulative increase at each time-point. In addition, compared to baseline, we found an increase in SpO_2_ values at post-prone 8th hour and later.

Prone positioning is labor-intensive and requires close monitoring. Although precautions are taken during prone positioning, complications are still reported, exemplified by various events, including an increased risk of unplanned extubation, endotracheal tube obstruction, displacement of the endotracheal tube, periorbital edema, facial edema, facial injury, and tracheal stenosis [1]. In the meta-analysis of Wright et al., it was reported that ventilation in the prone position was a safe method for patients in which ICP could be monitored [12]. However, another issue is that the literature reports different approaches and durations for the treatment of patients in the prone position. Current studies report that treatment in the prone position ranges from 4 h per day to 36 continuous hours or longer [13–16]. An ideal duration for the prone position has not yet been determined. However, Guérin and colleagues recommended a continuous prone position for at least 16 h, with sedation and neuromuscular blockade, in order to reduce mortality in ARDS [17]. In our study, patients diagnosed with ARDS who had a PaO_2_/FiO_2_ value of < 150 mmHg were placed in the prone position for 24 h using standard sedation and neuromuscular blockade, and intermittent ONSD measurements were made during treatment with a focus to determine whether prolonged prone positioning affects ICP. Our findings did not show an increase in ICP within 24 h following transition into the prone position. Kim JY et al. [18] showed a decrease in ONSD by decreasing EtCO_2_ (end-tidal CO_2_) values from 40 mmHg to 30 mmHg with short-term hyperventilation, and reported that ONSD can be altered by the changes in EtCO_2_. We included patients with a pCO_2_ value of 35–45 mmHg in our study. Two patients whose arterial blood pCO_2_ values were outside of the 35–45 mmHg range during follow-up were excluded. We believe that the consistency of ONSD values during our 24-hour study period is associated with the maintenance of stable ICP by appropriate anesthesia and ventilation in the prone position. Our results show that a possible increase in ICP can be prevented with proper management of patients in the prone position.

The most reliable method for evaluating ICP is direct measurement with an invasive monitoring device (e.g., extraventricular drainage). However, major complications such as infection and hemorrhage may develop with the use of invasive methods [19]. Although non-invasive techniques such as CT and MRI can be used, these techniques are expensive, require time to achieve results, and are not bedside methods. In addition, due to deep hypoxia in patients with ARDS, problems may be encountered during the transport of the patient, limiting accessibility. ONSD ultrasonography is a non-invasive, reproducible and bedside method. This technique can be lifesaving, especially when invasive ICP monitoring is contraindicated (e.g. due to coagulopathy) or when a specialist is not available for invasive monitor placement in the event of an emergency. There are studies using USG and ONSD measurements in cases that require rapid diagnosis and follow-up of ICP in intensive care units [20–22]. Normal ONSD ranges between 4.5 and 5.0 mm in healthy adults [23]. In their study involving post-cardiac arrest patients, Lee et al. [5] found better neurological outcomes in patients with ONSD values of < 5 mm. High ONSD was found to be associated with poor neurological prognosis and increased mortality in post-cardiac arrest patients by Chelly et al. [24]. The close relationship between ICP change and ONSD values has been demonstrated in the meta-analysis authored by Robba and colleagues [25]. In addition, the researchers also found that ONSD was correlated with ICU mortality. In a later study, Robba et al. [26] developed a formula for non-invasive measurement of ICP with the use of ONSD measurement (nICPONSD = 5 × ONSD − 14 [nICPONSD in mmHg, ONSD in mm]).

Although the evaluation of changes in ICP by USG-guided ONSD measurement appears to provide similar results to those obtained with invasive methods in many clinical studies, it has been reported that the measurement is operator-dependent, which is considered to be a weakness of the method [27, 28]. Despite the fact that the accuracy of USG-guided ONSD measurement is associated with the experience of the operator, Tayal et al. [29] stated in their studies that learning USG-guided ONSD measurement is easier than mastering the use of Doppler USG or assessing papilledema for the same purpose. Moretti et al. [30], stated that it would be sufficient to observe 10 normal and 3 pathological optic nerve diameters in order to gain sufficient experience to perform optic nerve USG. In our study, all ONSD measurements were made together by two experienced anesthesiologists who had received USG training. Additionally, instead of a single measurement during the post-prone period, we performed intermittent measurements over 24 h. We demonstrated that, with ONSD measurements, a safe and reliable follow-up can be achieved in patients ventilated in the prone position.

Studies reporting that prone positioning can prevent ventilator-induced lung injury are available in the literature [1, 16]. According to the World Health Organization (WHO) update on 25 January 2021, recommendations for ventilator settings have been found for the prone position, as well as lung-protective ventilation for ARDS patients (Low tidal volumes: 4–8 mL/kg estimated body weight and low inspiratory pressures: plateau pressure < 30 cmH2O) [31]. In the study by Guérin et al., the following were recommended: 6 mL/kg for estimated body weight, Pplateau < 30 mmHg, and appropriate PEEP selection [17]. In our study, we determined our ventilator settings similar to the lung-protective ventilation strategies reported by WHO and the research by Guérin and colleagues. In our study, we found improvement in oxygenation at all times during the 24-hour prone positioning in which lung protective ventilation strategy was accompanied by appropriate sedation and neuromuscular blockade.

### Limitations

There are some limitations in our study. First, it is not a blinded study as ONSD measurements were made by the same anesthesiologists during follow-up. However, since both anesthesiologists were experienced in the ONSD measurement technique, our study was planned with this plan in order to increase measurement accuracy. Secondly, this is not a randomized controlled trial, which prevented comparative analyses between different positions. Finally, despite the fact that our study has a power of 90% according to the described G-power analysis, the number of patients might be considered as being low for generalization.

## Conclusion

In conclusion, the current data shows that the prone position does not increase ICP within 24 h of transition. Therefore, in the context of ICP, mechanical ventilation in the prone position can be applied reliably –given that sedation is sufficient, neuromuscular blockade is performed, and mechanical ventilation is applied with appropriate strategies. Also, it appears that ONSD measurements can enable safe follow-up of patients ventilated in the prone position.

## Data Availability

The datasets used and analyzed during the current study are available from the corresponding author on reasonable request.

## References

[1] Guérin C, Albert RK, Beitler J, Gattinoni L, Jaber S, Marini JJ (2020). Prone position in ARDS patients: why, when, how and for whom. Intensive Care Med.

[2] Fernando SM, Ferreyro BL, Urner M, Munshi L, Fan E (2021). Diagnosis and management of acute respiratory distress syndrome. CMAJ.

[3] Guérin C, Beuret P, Constantin JM, Bellani G, Garcia-Olivares P, Roca O (2018). A prospective international observational prevalence study on prone positioning of ARDS patients: the APRONET (ARDS prone position network) study. Intensive Care Med.

[4] Lee JM, Bae W, Lee YJ, Cho YJ (2014). The efficacy and safety of prone positional ventilation in acute respiratory distress syndrome: updated study-level meta-analysis of 11 randomized controlled trials. Crit Care Med.

[5] Nekludov M, Bellander BM, Mure M (2006). Oxygenation and cerebral perfusion pressure improved in the prone position. Acta Anaesthesiol Scand.

[6] Reinprecht A, Greher M, Wolfsberger S, Dietrich W, Illievich UM, Gruber A (2003). Prone position in subarachnoid hemorrhage patients with acute respiratory distress syndrome: effects on cerebral tissue oxygenation and intracranial pressure. Crit Care Med.

[7] Roth C, Ferbert A, Deinsberger W, Kleffmann J, Kästner S, Godau J (2014). Does prone positioning increase intracranial pressure? A retrospective analysis of patients with acute brain injury and acute respiratory failure. Neurocrit Care.

[8] Frattalone AR, Stevens RD (2011). Intracranial pressure and its surrogates. Intensive Care Med.

[9] Moraes FM, Silva GS (2021). Noninvasive intracranial pressure monitoring methods: a critical review. Arq Neuropsiquiatr.

[10] Montorfano L, Yu Q, Bordes SJ, Sivanushanthan S, Rosenthal RJ, Montorfano M (2021). Mean value of B-mode optic nerve sheath diameter as an indicator of increased intracranial pressure: a systematic review and meta-analysis. Ultrasound J.

[11] Geng W, Chen C, Sun X, Huang S (2021). Effects of sevoflurane and propofol on the optic nerve sheath diameter in patients undergoing laparoscopic gynecological surgery: a randomized controlled clinical studies. BMC Anesthesiol.

[12] Wright JM, Gerges C, Shammassian B, Labak CM, Herring EZ, Miller B (2021). Prone position ventilation in neurologically ill patients: a systematic review and proposed protocol. Crit Care Med.

[13] Beuret P, Carton MJ, Nourdine K, Kaaki M, Tramoni G, Ducreux JC (2002). Prone position as prevention of lung injury in comatose patients: a prospective, randomized, controlled study. Intensive Care Med.

[14] Gattinoni L, Tognoni G, Pesenti A, Taccone P, Mascheroni D, Labarta V, Prone-Supine Study Group (2001). Effect of prone positioning on the survival of patients wifh acute respiratory failure. N EngI J Med.

[15] Papazian L, Gainnier M, Marin V, Donati S, Arnal JM, Demory D (2005). Comparison of prone positioning and high-frequency oscillatory ventilation in patients with acute respiratory distress syndrome. Crit Care Med.

[16] Parker EM, Bittner EA, Berra L, Pino RM. Efficiency of prolonged Prone Positioning for mechanically ventilated patients infected with COVID-19. J Clin Med. 2021;10. 10.3390/jcm10132969. 10.3390/jcm10132969 PMC8267703 34279453

[17] Guérin C, Reignier J, Richard JC, Beuret P, Gacouin A, Boulain T (2013). Prone positioning in severe acute respiratory distress syndrome. N Engl J Med.

[18] Kim JY, Min HG, Ha SI, Jeong HW, Seo H, Kim JU (2014). Dynamic optic nerve sheath diameter responses to short-term hyperventilation measured with sonography in patients under general anesthesia. Korean J Anesthesiol.

[19] Ross IB, Dhillon GS (2003). Ventriculostomy-related cerebral hemorrhages after endovascular aneurysm treatment. AJNR Am J Neuroradiol.

[20] Blaivas M, Theodoro D, Sierzenski PR (2003). Elevated intracranial pressure detected by bedside emergency ultrasonography of the optic nerve sheath. Acad Emerg Med.

[21] Jang T, Aubin C (2005). The use of serial ocular ultrasonography in the care of patients with head injury. Ann Emerg Med.

[22] Moretti R, Pizzi B (2011). Ultrasonography of the optic nerve in neurocritically ill patients. Acta Anaesthesiol Scand.

[23] Romagnuolo L, Tayal V, Tomaszewski C, Saunders T, Norton HJ (2005). Optic nerve sheath diameter does not change with patient position. Am J Emerg Med.

[24] Chelly J, Deye N, Guichard JP, Vodovar D, Vong L, Jochmans S (2016). The optic nerve sheath diameter as a useful tool for early prediction of outcome after cardiac arrest: a prospective pilot study. Resuscitation.

[25] Robba C, Santori G, Czosnyka M, Corradi F, Bragazzi N, Padayachy L (2018). Optic nerve sheath diameter measured sonographically as non-invasive estimator of intracranial pressure: a systematic review and meta-analysis. Intensive Care Med.

[26] Robba C, Donnelly J, Cardim D, Tajsic T, Cabeleira M, Citerio G (2019). Optic nerve sheath diameter ultrasonography at admission as a predictor of intracranial hypertension in traumatic brain injured patients: a prospective observational study. J Neurosurg.

[27] Kimberly HH, Shah S, Marill K, Noble V (2008). Correlation of optic nerve sheath diameter with direct measurement of intracranial pressure. Acad Emerg Med.

[28] Dubourg J, Javouhey E, Geeraerts T, Messerer M, Kassai B (2011). Ultrasonography of optic nerve sheath diameter for detection of raised intracranial pressure: a systematic review and meta-analysis. Intensive Care Med.

[29] Tayal VS, Neulander M, Norton HJ, Foster T, Saunders T, Blaivas M (2007). Emergency department sonographic measurement of optic nerve sheath diameter to detect findings of increased intracranial pressure in adult head injury patients. Ann Emerg Med.

[30] Moretti R, Pizzi B (2011). Ultrasonography of the optic nerve in neurocritically ill patients. Acta Anaesthesiol Scand.

[31] World Health Organization. COVID-19 clinical management: living guidance, 25 January 2021. https://apps.who.int/iris/handle/10665/338882. Accessed 25 Nov 2022

